# Australian Foster Carers’ Views and Concerns Regarding Maternal Drug Use and the Safety of Breastmilk

**DOI:** 10.3390/children8040284

**Published:** 2021-04-07

**Authors:** Stacy Blythe, Kath Peters, Emma Elcombe, Elaine Burns, Karleen Gribble

**Affiliations:** 1 School of Nursing and Midwifery, Western Sydney University, Penrith 2763, Australia; k.peters@westernsydney.edu.au (K.P.); k.gribble@westernsydney.edu.eu (E.E.); e.elcombe@westernsydney.edu.eu (E.B.); e.burns@westernsydney.edu.eu (K.G.); 2 Translational Research and Social Innovation (TReSI) Group, Ingham Institute of Applied Medical Research, Liverpool 2170, Australia

**Keywords:** infant feeding, breast milk, infant nutrition, survey, foster care, substance exposed infants

## Abstract

Parental substance misuse and mental health issues are major factors associated with infant placement into out-of-home care. Such placements may result in disruption and/or cessation of breastfeeding. Provision of breastmilk to infants in out-of-home care (OOHC) is desirable in terms of infant health and development, and also in supporting maternal caregiving. However, little is known about how breastfeeding is supported for infants in out-of-home care. This study used an online survey to explore the facilitation of breastfeeding in the context of OOHC and foster carers’ management of expressed breastmilk (EBM). Foster carers were generally open to the idea of maternal breastfeeding and infants in their care receiving EBM from their mothers. However, the majority of respondents expressed concern regarding the safety of EBM for infant consumption due to the possibility of harmful substances in the milk. Concerns regarding the safety of handling EBM were also prevalent. These concerns caused foster carers to discard EBM. Findings suggest foster carers’ may lack knowledge related to maternal substance use and breastmilk. Better integration between health care and social service systems, where the voices of mothers, foster carers and child protection workers are heard, is necessary to develop solutions enabling infants living in OOHC access to their mother’s breastmilk.

## 1. Introduction

Infants and young children (<4 years) represent the highest proportion of admissions into out-of-home care (OOHC) in many western countries, including Australia [1], the United States [2] and the United Kingdom [3]. In Australia, infants under one year of age are more likely, than other aged children, to be the subject of allegations and substantiations of child abuse and/or neglect and thus more likely to require OOHC services [1]. The majority of infants in OOHC reside in home-based care with foster carers (inclusive of kinship carers) who have been vetted and authorized by the relevant governing body to provide day-to-day care [1,2]. Parental substance use and mental health issues are two major factors associated with child protective service involvement and infant placement into OOHC [4]. 

Maternal substance use during pregnancy has multiple short- and long-term consequences for the unborn child. Short-term consequences include; poor fetal growth, microcephaly, congenital anomalies (e.g., cleft palate), and neurobehavioral abnormalities (e.g., increased irritability) [5,6]. Long-term consequences include; poor growth, impulsivity, attention deficits, language delay and academic underachievement [5,7,8,9]. Infants who are prenatally exposed to some substances, such as opioids, may also experience a withdrawal syndrome after birth, variously referred to as neonatal abstinence syndrome (NAS) and/or neonatal opioid withdrawal syndrome (NOWS). Symptoms may necessitate hospitalization and include; excessive crying, metabolic dysfunction, hypertonicity, as well as gastrointestinal and feeding difficulties [10]. Nurses and midwives report caring for infants with NAS and their families to be challenging [11].

The first two years of a child’s life represents a critical period of development and how infants and young children are fed can impact their life course [12]. Exclusive breastfeeding from birth until six months of age, and ongoing breastfeeding into the second year of life, are recommended for all children because this supports optimal health, growth, and development [13,14]. Premature cessation of breastfeeding is associated with increased risk of serious infectious disease requiring hospitalization, childhood obesity, type 2 diabetes, Sudden Infant Death Syndrome (SIDS), necrotizing enterocolitis (NEC), and impaired cognitive development [15,16]. These risks hold particular salience for children living in OOHC as they experience an elevated incidence of physical, mental, and developmental health issues compared to the general population [17,18,19]. In addition, breastfeeding supports maternal caregiving capacity and is protective against maternal neglect [20,21]. Provision of breastmilk to infants in OOHC is therefore desirable in terms of infant health and development and also in supporting maternal caregiving in case of reunification. 

In recognition of the importance of breastfeeding, a variety of international human rights documents outline rights held by mothers and children in relation to breastfeeding [20,22]. However, women use illicit substances or are on opioid maintenance programs have lower breastfeeding initiation rates than the general population [23,24]. Concerns related to the potential impact of substances on infants as well as factors associated with substance use such as low socio-economic status, poor nutrition, lack of education and mental health disorders can make enabling breastfeeding for the infants of women who use substances challenging [25,26]. There is a need to better support women’s infant feeding decision making in the context of their individual circumstances and specific substance use [27]. Women’s decisions regarding breastfeeding should be guided by well-informed health care providers who have specialist knowledge of both the importance of breastfeeding and potential harms of substance use while breastfeeding to both the infant and mother [25]. It has been argued that social workers have a responsibility to support infants access to breastmilk [20]. Although there is limited literature related to this topic, what is available suggests infants who have spent time in OOHC are less likely to be breastfed [28]. There is some evidence that women’s decisions to breastfeed or provide expressed milk to their infants living in OOHC is not well supported in Australia [29]. Nevertheless, in 2019, the Australian government and governments of each Australian state and territory agreed that both health and child protection services should, as a priority action, provide breastfeeding support to mothers and infants interacting with the child protection system [12].

As providers of day-to-day care, foster carers have a pivotal role in ensuring proper infant nutrition. However, little is known about foster carers’ practices around mother’s’ decision to breastfeed or provide breastmilk to their infant in OOHC. To our knowledge, there have been no prior studies exploring the facilitation, or not, of ongoing breastfeeding in the context of OOHC or foster carers’ management of expressed breastmilk (EBM) provided by mothers. This study sought to fill that gap. 

## 2. Materials and Methods

The results reported here are drawn from a larger, mixed-methods study aimed at exploring practices related to infant feeding in the context of foster care in Australia. The study employed a descriptive, online survey containing both closed- and open-ended questions regarding infant feeding practices for foster carers and individual interviews with foster carers, as well as other key stake holders (e.g., case workers). A concern regarding maternal substance use, and the incidence of prenatal substance exposure arose naturally from the data. The findings reported in this paper are from the online survey. 

### 2.1. Survey

The online survey was developed by the researchers (KG and SB) and hosted by the online survey platform, Qualtrics^®^. In addition to their academic positions, KG is an Australian Breastfeeding Association counsellor, community educator, and advisor and an authorized foster carer and SB is an authorized foster carer with firsthand experience of infant feeding in OOHC and caring for substance exposed infants. Survey questions were based on extant literature related to infant nutrition and explored issues related to participants’ experiences feeding infants in OOHC. The interactive nature of the survey allowed for participants to answer only questions that were relevant to their infant feeding experience. For example; participants were asked if expressed breastmilk (EBM) had been provided by mothers for infants in their care. If the answer to this question was ‘no’, then no further questions regarding that scenario were asked. Each participant could have been asked a maximum of 102 questions over 9 segments. Approximately 80% of these questions were multiple choice or ‘tick a box’. Participants were encouraged to answer all relevant questions, and prompted to do so if they initially chose to skip a question. However, participants were also able to ignore prompts and move on to further questions. Both open and closed-ended questions were used in the survey in order to obtain as much information about participants’ experiences as possible. Demographic information related to sex, age, education, and state/territory of residence were collected as well as details regarding the length of time and types of foster care respondents had provided. The survey was piloted prior to circulation to ensure questions were deemed relevant and easy to understand. 

The survey was shared on Facebook, Twitter and LinkedIn sites associated with the School of Nursing and Midwifery at Western Sydney University as well as several closed Facebook sites specific to foster carers in Australia. Respondents were encouraged to share the survey link with other potential participants.

### 2.2. Participants

This survey was open to authorized foster carers who had experience caring for an infant (0–12 months old) in an Australian jurisdiction within the last five years were eligible to participate in the study. Survey recruitment via social media occurred between February and June 2018. 

### 2.3. Ethical Considerations

The Human Research Ethics Committee of Western Sydney University granted ethics approval for this study [H12400]. An explanation of the study’s intent and the voluntary nature of research was described before the first survey question. Completion of survey questions was deemed consent to participation. 

### 2.4. Data Analysis and Management

Data from the survey was password protected and stored within Qualtrics^®^ and on an Excel^®^ spread sheet in CloudStor^®^. Analysis was completed using IBM^®^ SPSS Statistics v25.0.0.1 with descriptive statistics (including sample size, mean, median and percentage), and chi squared analyses were used for closed-ended survey questions. 

## 3. Results

There were a total 227 surveys recorded. A total of 184 surveys were included in the analysis. Surveys were excluded from the analysis if they were incomplete and from a repeated IP address (*n* = 7), if their survey duration was under 2 min (*n* = 23), or if the primary outcome questions were not answered (*n* = 13). The average number of questions answered per survey was 53.5, with a median survey completion duration of 14 min.

### 3.1. Demographics

Study participants were from all Australian states and territories. The majority of survey participants were female (97%), aged 40–49 years (41%), and had a post-secondary school education (66%) (Table 1a). More than a quarter of the sample (27%) indicated they had no biological children of their own and so had not had day-to-day care of an infant before becoming a foster carer.

Respondents reported 1 to 15 years of experience providing foster care to infants, with one to three years (32%, *n* = 60) and five to ten years (25%, *n* = 46) being the most common durations reported. The number of infants for whom participants had cared over their total fostering experience varied widely. Nearly one quarter (23%, *n* = 43) of respondents had provided care to only one infant, however, over one third of respondents (38%, *n* = 70) reporting having cared for five or more infants (Table 1b). One participant estimated 80–100 infants had been in her care. The majority of carers (85%) reported having multiple types of foster care authorization (range: 1 to 6, median: 4); long-term (80%, *n* = 148), short-term (84%, *n* = 154), and crisis care (71%, *n* = 130) were the most common. 

In total, foster carers had cared for more than 1155 infants. Of these, 71% were under six months when placed in their care and 40% were placed into their care directly from hospital as newborns (Table 1b).

### 3.2. Support of Breast Feeding in the Foster Care Community

When asked “Are you supportive of the infant having frequent contact with their mother to enable breastfeeding?” 40% responded “yes”, 49% responded “Unsure” and less than 10% responded ‘No’. Sixty-three percent of foster carers (*n* = 117) had first-hand breastfeeding experience (i.e., they breastfed their own children). Foster carers with personal breastfeeding experience were more supportive of frequent contact for breastfeeding, however this difference was not statistically significant (*chi.sq.* = 5.56, *df* = 2, *p* = 0.062). Despite this generally positive and supportive view of infants in OOHC being breastfed, foster carers described several concerns, most significantly in relation to the safety of the breastmilk for infant consumption and potential for the presence of harmful substances in the milk. 

### 3.3. Breast Milk Safety

Eighty-five percent of respondents reported concerns regarding whether breastmilk provided by mothers was safe for infants. Post hoc analysis showed that this did not differ between foster carers with or without breast feeding experience, nor did it differ between foster carers who had (*n* = 47), or had not (*n* = 137), received EBM for an infant in their care. 

**Figure 1:**
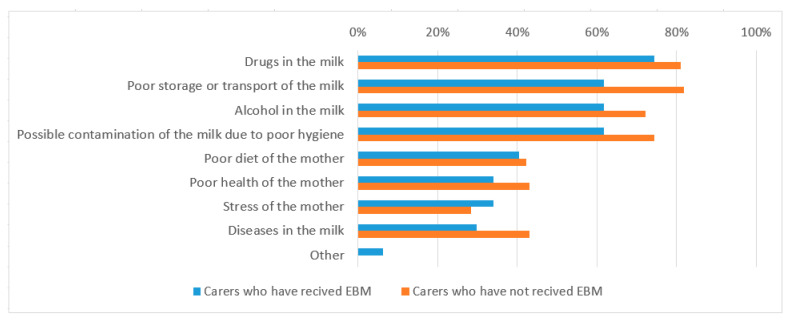
Percentage of carers concerned about the safety of consuming supplied breastmilk.

The most frequent concern reported by foster carers was the possibility of harmful substances in the milk (79%). Concerns regarding alcohol in the milk, poor storage and transport of the milk, and possible contamination of the EBM due to poor hygiene were highly prevalent. Additional concerns regarding the health (30%) and wellness (42%) of the mother were also reported by responders (Figure 1).

**Figure 2:**
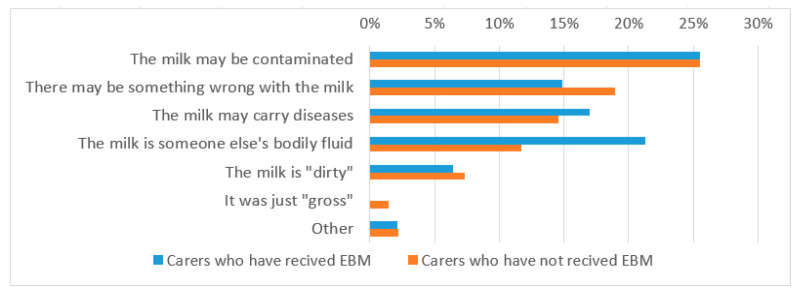
Percentage of carers concerned about handling of someone else’s breast milk.

Foster carers were also asked whether they had concerns about the safety of handling EBM from mothers, overall 31% of carers said they did have/would have concerns. As above, this did not vary with breast feeding experience or with whether they had received EBM for a child in their care. The most common concern in relation to handling milk was that it might be contaminated (26%) (Figure 2).

### 3.4. Behaviours as a Result of Breast Milk Saftey Concerns

Foster carers’ response to concerns about the safety of EBM provided to them was split. Some foster carers fed the EBM to the infant despite their concerns, while others discarded it. Some foster carers reported both feeding and discarding EBM. In relation to this matter, foster carers who had not received EBM were presented with a hypothetical scenario where EBM was provided for an infant in their care, and asked what they would do. Their responses different significantly from foster carers who had received EBM. For instance, 23% of foster carers who had received EBM reported that they fed it to the infant despite their concerns whereas in the hypothetical situation, only 5% of foster carers said they would do this. In addition, a significant proportion of foster carers who had not received EBM reported ‘other’ as their response. Post hoc categorisation of these related comments showed that 25 (90%) of these foster carers would seek further information prior to making a decision (Table 2).

In total, 80% (*n* = 31) of concerned foster carers who had received EBM indicated they had discarded breastmilk, with 97% (*n* = 30) of these respondents discarding the breastmilk due to safety concerns. Of those participants who had not yet been provided EBM (*n* = 137), 42% (*n* = 57) indicated their concerns would cause them to discard the breastmilk rather than give it to the infant.

### 3.5. Carer Training Regarding EBM

Overall, 14% of foster carers reported they received training related to infant feeding and nutrition from their foster care agency. This training related to correct selection, preparation and storage of infant formula; bottle feeding technique; and common feeding problems. A small number of those foster carers (*n* = 7) also reported receiving training in bottle feeding a baby used to breast feeding and/or how to safety store and prepare EBM. While this study did not specifically look at feeding babies with NAS, none of the foster carers reported receiving information or training related to the safety of handling EBM.

## 4. Discussion

Foster carer concerns regarding the potential for infant exposure to harmful substances via breast milk consumption emerged as a barrier to the provision of breastmilk to infants in OOHC. Concerns around the safety and handling of EBM were also prominent. We propose that foster carers’ lack of information about the background of infants placed with them and a knowledge deficit regarding breastfeeding, and in particular, substance use and breastfeeding, underlay these barriers. Integrated care between the health and child protection systems is necessary to facilitate infants’ access to safe breast milk. 

### 4.1. Lack of Background Information

In general foster carers were supportive of infants in OOHC being breastfed or receiving EBM from their mothers. However, the vast majority also held concerns regarding the safety of breastmilk for infant consumption. Although, this study did not solicit information from foster carers regarding why the infants placed with them had entered OOHC, their responses indicate that many were concerned about maternal substance use. It is in fact, probable that many respondents would not have information about whether infants had been prenatally substance exposed, born substance-dependent, or had experienced substance withdrawal. Even if they were aware of maternal substance use, they may not have been aware of what substances mothers had used. Poor communication between foster carers and social service professionals is a persistent problem repeatedly reported in the literature [30,31,32]. In a recent study of 1095 foster carers, less than half (*n* = 456, 46%) reported receiving sufficient background information when a child was placed into their care [33]. This lack of background information places foster carers in a milieu where informed decision-making regarding EBM use is not possible. In this study, the majority of foster carers seemed to believe the best action was to not provide infants in their care with breastmilk from their mothers *just in case* it was unsafe. Unfortunately, this likely means that some infants were denied access to safe breastmilk. 

### 4.2. Knowledge Deficit Regarding Breast Milk Safety

In this study, foster carers expressed concerns about handling breastmilk because it was a bodily fluid. Fear of accidentally touching breastmilk or even handling containers of breastmilk is a concern that has been manifest in contexts outside of OOHC. For example, women in the workplace report facing resistance to storing EBM in shared work refrigerators [34]. Childcare workers have communicated concerns about handling and feeding EBM. Schafer and colleagues found that childcare workers in the United States felt uncomfortable handling breastmilk and were encouraged to wear gloves when preparing and feeding EBM (but not infant formula) [35]. Research with Australian childcare workers revealed a similar attitude towards handling EBM with infant formula feeding positioned as the ‘normative’ feeding method [36]. These concerns and practices are unfounded with no documented cases of disease transmission via EBM handling, and with human breastmilk not being classified as a bodily fluid that requires universal precautions for handling [37]. In OOHC, education and training about breastfeeding may support mothers to provide EBM for their infant and enable foster carers to feel comfortable and confident about handling EBM [35,38,39]. Research from the child care environment demonstrates that support for EBM feeding can facilitate breastfeeding continuance by mothers [36,40].

One of the concerns raised by foster carers in this study was the potential risk of harm to infants if EBM contained substances that could be harmful to them such as medications, illicit drugs, or alcohol. Given that infants commonly enter OOHC for reasons related to parental substance use or mental health challenges, this is a reasonable concern [4]. However, the risk of harm to the infant due to substances in breastmilk needs to be weighed against the overall value of breastmilk to infant health and development and the risks associated with feeding infant formula [41]. The care the mother is demonstrating by breastfeeding and/or providing EBM should also be considered. 

Both prescription and illicit drugs consumed by women may be present in breastmilk although, for most substances the amount is likely to be small with the concentration reducing over time as the drug is metabolized [42]. Factors that impact the level and significance of infant substance exposure via breastmilk include: the substance involved, the timing of maternal substance intake in relation to breastfeeding or milk expression; the volume of milk ingested by the infant; and the age of the infant; with those under two months at greater risk than those over six months of age [42]. 

The impact of substances in breastmilk on infants will vary depending on the drug involved. Although most drugs are present in breastmilk, to some degree, the majority are not a concern in breastfeeding [42], this includes many psychiatric medications [43]. Harm minimization strategies can further assist in reducing infant exposure to substances [44]. This may include reducing substance use, prescription of a medication more compatible with breastfeeding or expressing milk before ingesting substances [42,43,44,45].

By and large, only women who are actively using illicit substances and are not engaged in a substance abuse treatment program should be discouraged from breastfeeding or providing EBM [46]. Conversely, women who are engaged in a substance abuse treatment programs are generally encouraged to breastfeed, as the associated oxytocin release may aid in recovery and prevent relapse [47]. This is particularly relevant for recovering mothers with a history of opioid use whose infants have NAS. Breastfeeding in this context has been found to decrease incidence and severity of infant withdrawal symptoms, reduce infant need for pharmacotherapy and shorten the length of hospital stay [48]. Decisions regarding whether breastmilk is safe for an infant requires comprehensive individual assessment. In Australia, health services to assist in such assessment exist and are available to anyone including mothers, health professionals, and would also be available to child protection workers, e.g., [49]. We would argue when an infant is residing in OOHC, the determinations of such assessments should not only be shared with the mother, but also communicated to the foster carer and child protection worker. 

### 4.3. Need for Integrated Care for Infants Entering OOHC

During pregnancy and antenatally through the health care system, women are provided with education and anticipatory guidance on the care of their infants. Provision of this support is part of the role of midwives [50]. This education covers aspects of infant care including bathing, normal infant behaviour, safe sleep, and infant feeding. Education on breastfeeding is standard for all women while education on safe practice in formula feeding is provided where mothers have chosen to use infant formula. Where infants have special medical needs, for example a cleft palate or an intellectual disability, mothers will be provided with specialised support regarding their needs [51,52]. Mothers of infants with NAS, are also given additional support in understanding their infant’s special needs, including how to interpret their behaviour and comfort them [53,54,55]. 

However, as discussed, when an infant enters OOHC, their foster carer may receive very little information about their health needs or background. In many instances, even when infants enter care shortly after birth, there is no contact between the foster carer and hospital staff, as it is common for infants to be delivered to foster carers by child protection workers [56]. Thus, even though many infants entering OOHC would be considered to have special medical needs, including infants who have been substance exposed, foster carers may receive no information or support from the health care system to assist in the infant’s care. This is particularly concerning when foster carers may have no previous infant care experience which may be common; more than one quarter of foster carers in this study did not have biological children. While child protection workers may be able to provide some information and support, it is likely they do not have the specialised medical and health knowledge needed to provide adequate support [57]. This lack of knowledge and support is evident in foster carers’ management and concerns regarding EBM. 

It has long been recognized that the inclusion of multiple health and social disciplines is essential for optimum outcomes for populations who require both health and social care [58,59]. This integrated care is acknowledged as being particularly important for people with complex care needs and those who are vulnerable [58,60]. This would include infants who have entered OOHC, particularly those with medical conditions such as NAS. The negative consequences of not providing integrated care for these infants are far-reaching into adulthood and beyond, and include substantial physiological, psychological, social and financial impacts [5,7].

In an attempt to address health inequities and improve health outcomes for vulnerable populations, Australia’s long-term health plan includes six integrated care programs, one of which focuses on vulnerable families [60]. Despite this well-intentioned plan, there are several barriers to the implementation of integrated care, resulting in a gap between health and social services. In order to ensure integrated care for these vulnerable infants, greater collaborations and effective information sharing across sectors and between key stakeholders, including foster carers, are required [61]. To successfully facilitate integrated care for infants with NAS, this would ideally be organized and enacted prior to their discharge from hospital and facilitated for the duration of their time in OOHC. In regard to breastfeeding and provision of EBM to infants in OOHC, effective, integrated, and continuing health system support needs to be provided to mothers, child protection workers, and foster carers. This is necessary to ensure appropriate risk management and mitigation and the safe continuance of breastfeeding and provision of EBM where ever possible. 

### 4.4. Limitations and Suggestions for Future Research

This study did not specifically investigate care of substance exposed infants, however, foster carers concern related to the potential for infant exposure to harmful substances via breastmilk consumption was apparent. Future research exploring foster carers’ experiences of caring for substance exposed infants is warranted. This research aimed to gain an understanding of foster carer support for breastfeeding and the provision of EBM in the out-of-home care context. It is important to acknowledge that the survey did not separate out the perspectives of kinship carers. It is possible that kinship carer experiences differ to other foster carers, given most have a pre-existing relationship with the infant’s parents. This study used social media to recruit research participants, a method which has gained momentum as it can facilitate easy access to some hard to reach populations, however it is not without its limitations and necessarily excludes individuals who do not use social media potentially biasing the sample [62]. 

Hearing the voices of all key stakeholders is essential if effective solutions are to be developed thereby enabling infants living in OOHC access to their mother’s breastmilk. Subsequent studies should investigate the experiences of mothers and case workers in order to gain a better understanding of the whole context. Finally, while this paper provides an Australian perspective, an increased incidence of infants with NAS [63,64] and concerns regarding infant access to breastmilk or breastfeeding in OOHC have also been reported in the UK and Canada [20,65]. We believe the issues articulated in this paper will resonate with other OOHC settings. 

## Figures and Tables

**Table 1:** (**a**): Demographic characteristics. (**b**): Number of infants ever fostered by study participants.

Characteristic		*n* (%)
(**a**)		
Gender	Female	178 (96.7%)
	Male	6 (3.3%)
Age	20–29 years	12 (6.5%)
	30–39 years	50 (27.2%)
	40–49 years	75 (41.3%)
	50–59 years	34 (18.5%)
	60–69 years	10 (5.4%)
	70+ years	2 (1.1%)
Highest level of education completed	Primary School	3 (1.6%)
	Secondary School	29 (15.8%)
	TAFE Qualification *	67 (36.4%)
	Bachelor’s Degree	61 (33.2%)
	Master’s Degree	7 (3.8%)
	Doctoral Degree	3 (1.6%)
	Other	14 (7.6%)
Duration of fostering experience	1–3 years	60 (32.6%)
	4–5 years	38 (20.7%)
	5–10 years	46 (25.0%)
	10–15 years	18 (9.8%)
	15+ years	22 (12.0%)
State or territory where fostered ^	New South Wales	49 (26.6%)
	Western Australia	42 (22.8%)
	Victoria	29 (15.8%)
	South Australia	26 (14.1%)
	Queensland	20 (10.9%)
	Australian Capital Territory	12 (6.5%)
	Northern Territory	8 (4.3%)
	Tasmania	4 (2.2%)
Type/s of foster care authorization ^	Long term	148 (80.4%)
	Short Term	154 (83.7%)
	Crisis/Emergency	130 (70.7%)
	Respite	115 (62.5%)
	Specialised care of infants	44 (23.9%)
	Other	20 (10.9%)
(**b**) ^&^		
Number of children under 12 months ever fostered	One	43 (23.2%)
	Two	27 (14.6%)
	3 or 4	39 (21.1%)
	5 to 9	42 (22.7%)
	10 to 19	15 (8.1%)
	20 or more	13 (7%)
Number of infants placed with foster carers as newborns directly from hospital	None	50 (27%)
	One	56 (30.3%)
	Two	29 (15.7%)
	3 or 4	22 (11.9%)
	5 to 9	13 (7%)
	10 to 19	5 (2.7%)
	20 or more	4 (2.2%)
Number of infants under six months of age at placement with foster carers	None	10 (5.4%)
	One	52 (28.1%)
	Two	27 (14.6%)
	3 or 4	37 (20%)
	5 to 9	35 (18.9%)
	10 to 19	11 (5.9%)
	20 or more	7 (3.8%)
	Total	179 (96.8%)

^ Carers were asked to select all that apply. * TAFE equates to post-secondary non-tertiary education according to the International Standard Classification of Education (ISCED). ^&^ Note: One carer did not answer this question and four provided non-specific or non-numeric answers such as ‘over 50′, and ‘numerous’. These five have not been included in part b of this table.

**Table 2:** Foster carers’ actual or planned response to concerns about the safety of expressed breastmilk (EBM) ^.

	Concerned Foster Carers Who Had Received EBM (*n* = 47)	Concerned Foster Carers Who Had Not Received EBM (*n* = 137)
Feed it to the infant anyway	9 (23%)	2 (5%)
Keep the milk, but do not give it to the infant	6 (15%)	41 (35%)
Discard the milk	31 (80%)	57 (48%)
Other	2 (5%)	28 (24%)

^ Carers were asked to select all that apply.

## Data Availability

The data presented in this study are available on request from the corresponding author. The data are not publicly available due to privacy restrictions.
